# Supplementary material for: Evidence for allocentric boundary and goal direction information in the human entorhinal cortex and subiculum
Source: Nat Commun. 2019 Sep 5;10:4004. doi: 10.1038/s41467-019-11802-9 (PMC6728372; doi:10.1038/s41467-019-11802-9)
Supplement: Supplementary file 1 — Supplementary Information [file 41467_2019_11802_MOESM1_ESM.pdf]

Evidence for allocentric boundary and goal direction information in the human entorhinal cortex and subiculum

Shine et al.

Supplementary Note 1

Although accuracy was matched across different allocentric boundary and goal directions, there was a significant effect in reaction times (RT) (Supplementary Figure 1). To check whether the linear SVM classifier was sensitive to this information, we carried out six binary classification models (i.e., North versus South (NvS), North versus East (NvE), North versus West (NvW) etc.), for each ROI. The resulting decoding accuracies were submitted to a repeated-measures ANOVA comprising the factors ROI (EC, subiculum) × Portion (Anterior, Posterior) × Model (NvS, NvE, NvW, SvE, SvW, EvW), which did not reveal any significant main effects or interactions [Repeated-measures ANOVA: all *F*s < 2.13, *p*s > 0.15]. If the classifier were detecting differences in RT then the main effect of Model would be of particular importance, however there was no evidence that this factor modulated decoding accuracy [Repeated-measures ANOVA: F (5, 135) = 0.94, p = 0.46, η_p^2 = 0.033]. To test more thoroughly any ROI-specific effects, pair-wise t-tests were carried out for the models most likely to show an effect driven by RT. Specifically, in line with the RT data, one might predict that the decoding accuracy of North versus West should be greater than for North versus South, given that North and West show the greatest disparity in RT. Consistent with the ANOVA, however, there was no evidence that decoding accuracy was higher for NvW relative to NvS in any of the EC or subiculum ROIs [Paired-sample t-test: all *t*s < 1.08, *p*s > 0.58].

Supplementary Note 2

As can be seen in Supplementary Figure 2, even when using eroded masks (see below) our effects of interest in the EC and subiculum remained consistent with the analysis in which the whole ROIs were used. Outside of our key ROIs (the EC and subiculum), it was possible to decode allocentric goal and allocentric boundary direction in the PHC (Supplementary Figure 3); decoding accuracies in the CA1 and CA23/DG did not survive Bonferroni correction (p = 0.008). Consistent with our previous control analysis, we also eroded these additional medial temporal lobe ROIs and found that decoding accuracy was reduced meaning that the effects in PHC were no longer significant (Supplementary Figure 4).

Supplementary Note 3

Consistent with previous research, we used an "Add" model[4] to capture the peak of the haemodynamic response function (HRF) relating to the stationary period of our trial. Although in the egocentric conditions we demonstrated that EC and subiculum do not appear to code for stimuli even when there are strong lower-level visual cues, we wanted to test further whether visual information could still contribute to the decoding performance observed in the EC and subiculum. To explore this possibility further, we examined decoding accuracy as a function of the portion of trial used for the analysis. Our prediction was that if the decoding performance reflects the coding of visual information, taking the portion of the trial corresponding to the peak of the HRF after the viewing of the passive movement should result in higher decoding accuracies relative to later portions of the trial. As can be seen in Supplementary Figure 5, allocentric

boundary decoding accuracy was not at its highest in posterior EC or subiculum in the 2-8s portion of the trial, which one would predict if the effect was driven by the visual properties of the stimuli. The same is true of allocentric goal decoding in anterior EC and subiculum.

Supplementary Note 4

Recent evidence in non-human animals has highlighted the neural underpinnings of egocentric goal and boundary representations. Specifically, goal vector coding has been demonstrated in the CA1 of bats[1], and both egocentric goal and boundary representations have been reported in the rodent anterior-lateral EC[2]. Unlike the allocentric boundary and goal conditions in our study, the egocentric conditions contain visual confounds. For example, in the egocentric boundary condition, the different classes comprise the boundaries to the participant's left, right, or directly in front of them. Similarly, for the egocentric goal condition, although the boundary position changes over trials, the position of the cue object will either be to the left or right of the participant. Consequently, one must be cautious with any interpretations regarding these analyses as they could be confounded by these lower-level visual properties. For completeness, we present in Supplementary Figure 6 the results of the egocentric decoding for the EC and subiculum. Similarly, we performed the same analysis for the other medial temporal lobe regions. In addition, given that the strong visual confounds of the analysis, we used a Freesurfer segmented mask of V1 to examine whether we could decode this information in visual regions (Supplementary Figure 7). Despite the strong visual confounds, it was

possible to decode egocentric goal location only in the anterior subiculum. That visual information was not strongly coded in the EC and subiculum is consistent with studies demonstrating that these regions are not sensitive to purely visual information[3]. In line with the strong visual confounds for the egocentric conditions, it was possible decode both properties in V1, as well as in CA23/DG. Furthermore, there was evidence that CA1 may contain information also regarding egocentric boundary direction.

Although we cannot rule out entirely the contribution of visual information to the decoding performance in EC and subiculum, we feel that (1) the pattern of decoding accuracy that shows decoding accuracy is not at its highest in the portion of the trial containing the putative peak of the HRF after visual input, (2) the inability to decode egocentric conditions with strong visual confounds, and (3) evidence from previous studies demonstrating that the EC/subiculum is insensitive to visual information[3], suggest that our effects are not simply an artifact of visual processing.

Supplementary Note 5

An alternative method used to examine multivariate patterns in the brain is representational similarity analysis (RSA)[5]. Unlike linear SVM classifiers in which the decision hyper plane separating different stimulus categories is supported by the most informative subset of voxels[6], RSA analyses take into account the representational structure across all voxels in an ROI. The beta estimates associated with different conditions are correlated, using for example Pearson's r, to examine the degree of similarity between the elicited responses.

Given that boundary vector/border cells are insensitive to boundary identity, we carried out an RSA to investigate the degree of similarity between responses associated with different boundaries that shared the same allocentric boundary direction versus those in which the allocentric boundary direction differed. Consistent with our decoding analysis, we created averages of the trial estimates across the three runs but for this analysis we averaged by allocentric boundary direction for each individual boundary. This created eight different conditions (four boundaries*two sides) in which we could compare boundary-specific, allocentric boundary direction representations. We then correlated the response associated with the same allocentric boundary direction across the two different boundaries (e.g., boundary 1-North with boundary 2-North etc.) for voxels in our EC, subiculum, CA1, CA23DG and PHC ROIs. To test for the degree of similarity in these responses, we Fisher z-transformed the correlation coefficients and compared the magnitude of these correlations relative to the different allocentric boundary direction across the two boundaries (e.g., boundary 1-North with boundary 2-South etc.). This had the effect of controlling for any possible visual confounds of the boundary texture whilst contrasting the allocentric boundary direction. As can be seen in Supplementary Table 1, there was significantly greater similarity in posterior EC for trials in which the allocentric boundary direction was the same versus those in which it differed. There was no evidence of increased similarity associated with allocentric boundary direction in any of the other ROIs.

Supplementary Note 6

During the fMRI scanner task, the on-screen positions of the landmarks in the forced-choice response were randomly assigned (either left, middle, or right). This was important for the decoding analysis, so that no other information confounded the spatial property that we were trying to decode. To ensure that there was no systematic bias in the position of these items, for each landmark we calculated the proportion of times it was located in the left, middle, or right position on the screen (Supplementary Figure 8). These values were submitted to a repeated-measures ANOVA, comprising the factors Landmark (Mountain, Cathedral, Clock tower, City) × fMRI run (1, 2, 3) × Position (left, middle, right) and revealed that there were no significant main effects or interactions [Repeated-measures ANOVA: all Fs < 1.44, ps > 0.20] relating to the spatial position of the items on screen.

For the decoding analyses, we used anatomical ROIs of the hippocampal subregions, comprising EC, subiculum, CA1, CA23/DG, and PHC traced manually on individual participant's T2-weighted images. Advanced Normalisation Tools was used to move these anatomical ROIs to EPI space, using a composition of the EPI-to-T1 inverse, and T2-to-T1, matrices (Supplementary Figure 10). The EC and subiculum ROIs into anterior and posterior portions by dividing them at the midpoint along their longitudinal axis. For our seven bilateral ROIs (bilateral posterior EC, anterior EC, posterior subiculum, anterior subiculum, PHC, CA1, CA23/DG) we assessed the mean temporal signal-to-noise ratio (calculated by dividing each voxel's mean intensity per run by its standard deviation over time and averaging the resulting

values across each ROI) and volume (Supplementary Figure 11). Although by using unsmoothed data we attempted to mitigate any leakage of our effects of interest between anterior and posterior sections, it is possible that in the EC and subiculum information overlapped between the posterior and anterior sections. To provide a more stringent test of the posterior-anterior distinction, we performed a control analysis in which we removed the influence of adjacent structures in the medial temporal lobe by eroding our ROIs to remove the outer layer of voxels from each region using FSL maths (box kernel = $1 \times 3 \times 1$ voxels) (Supplementary Figure 12).

Supplementary Figures

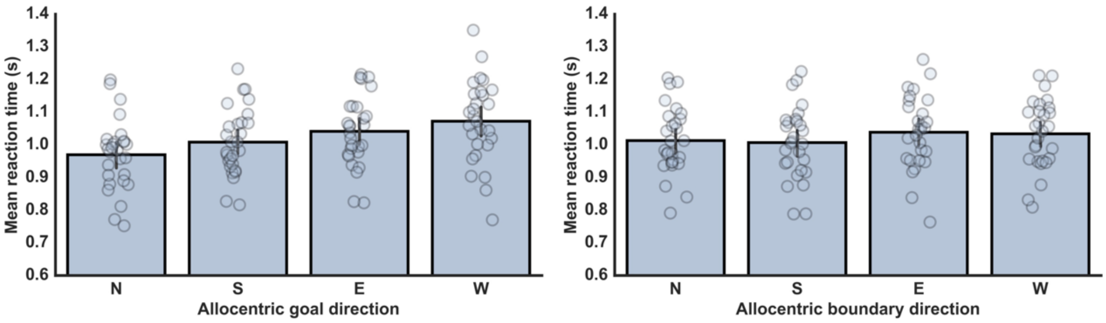

Supplementary Figure 1. Reaction times for the fMRI scanner task with trials coded separately according to allocentric goal and allocentric boundary direction. Individual subject's data points are represented by grey circles. Error bars represent 95% CI. Source data are provided as a Source Data file.

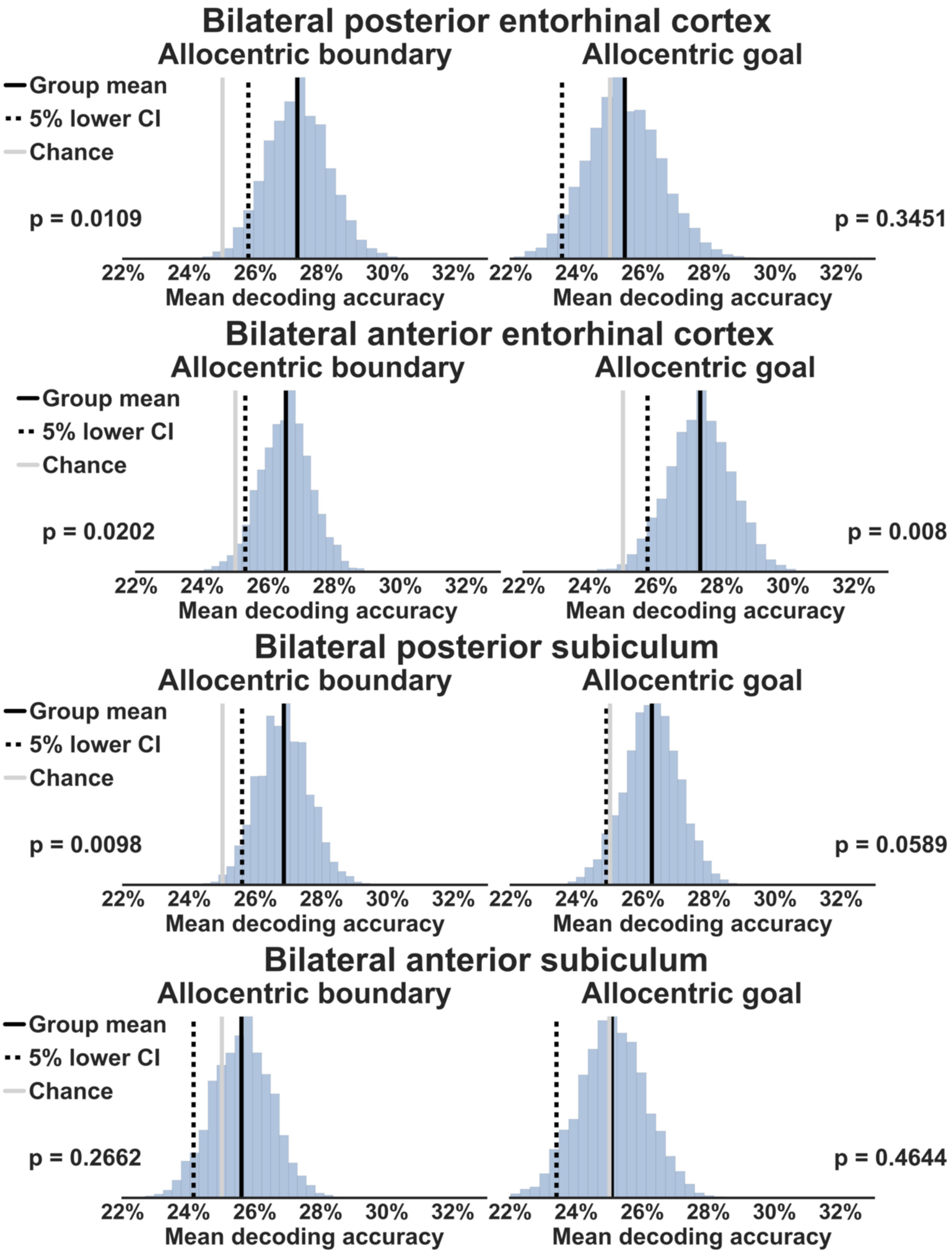

Supplementary Figure 2. fMRI decoding results in bilateral EC and subiculum using eroded masks. The results remained consistent even when using more conservative masks to remove the effect of neighbouring cortical regions. The only difference was that allocentric goal decoding in the anterior subiculum was no longer significant. All *p*-values were determined via non-parametric Monte Carlo significance tests. Source data are provided as a Source Data file.

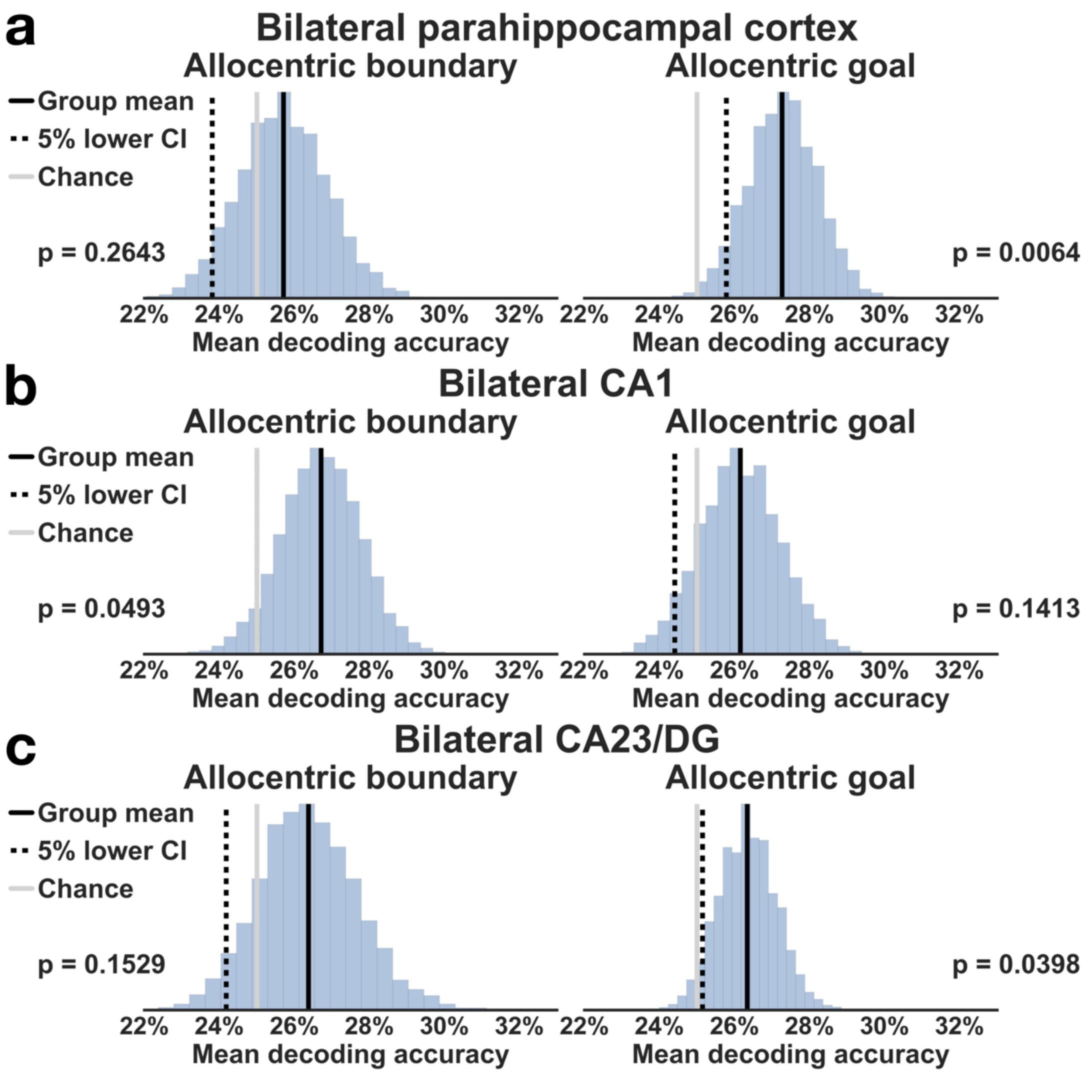

Supplementary Figure 3. Decoding of allocentric boundary and goal direction outside the EC and subiculum. (a) In the parahippocampal cortex, it was possible to decode allocentric goal direction, (b) but group decoding accuracies for allocentric boundary and goal direction in bilateral CA1, and (c) CA23/DG did not survive Bonferroni-correction ($p = 0.008$). All p-values were determined via non-parametric Monte Carlo significance tests. Source data are provided as a Source Data file.

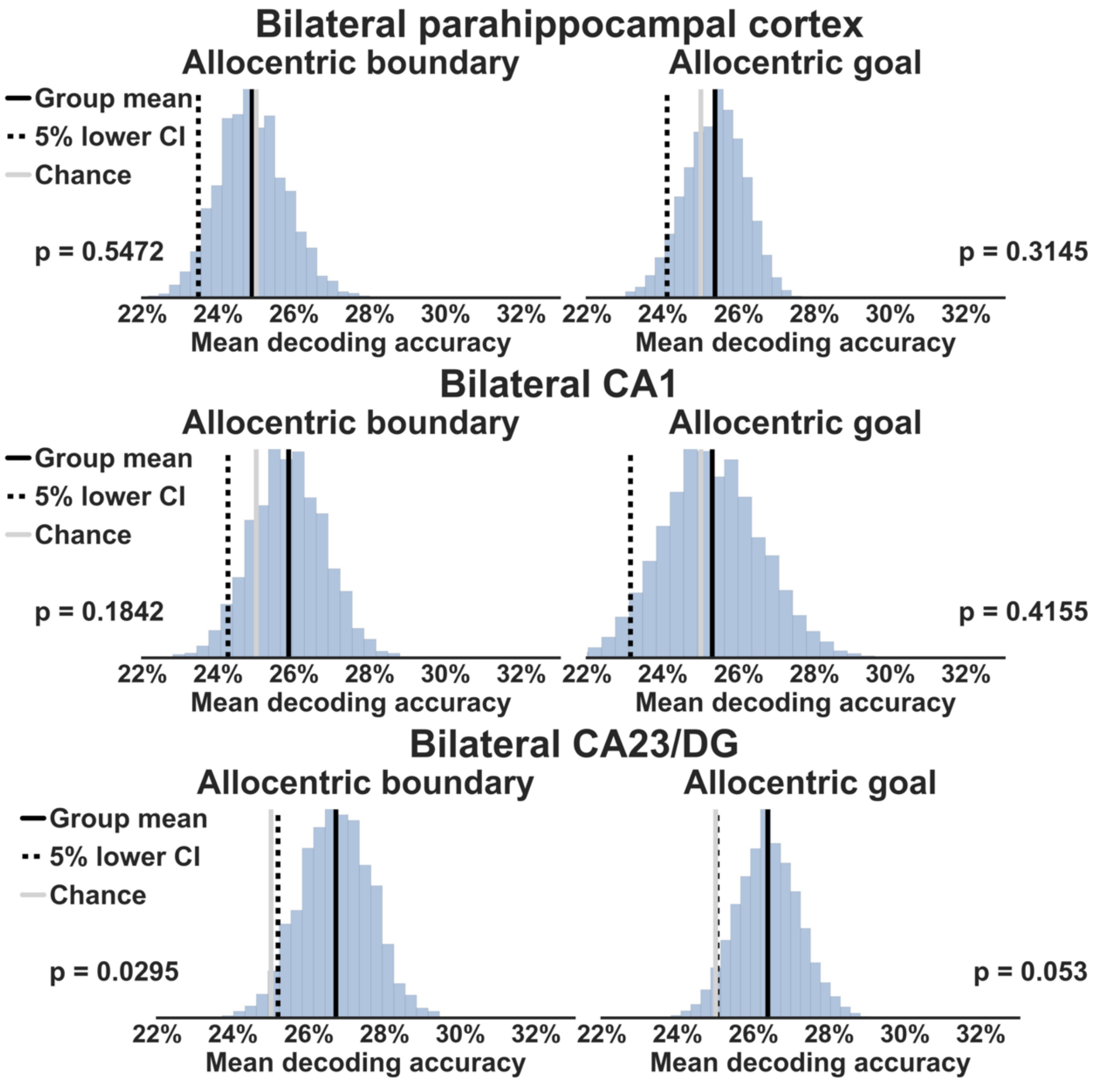

Supplementary Figure 4. Decoding of allocentric boundary and goal direction outside the EC and subiculum using eroded masks. (a) In the PHC, it was no longer possible to decode allocentric goal direction, and in no other regions did effects survive Bonferroni-correction (*p* = 0.008). All *p*-values were determined via non-parametric Monte Carlo significance tests. Source data are provided as a Source Data file.

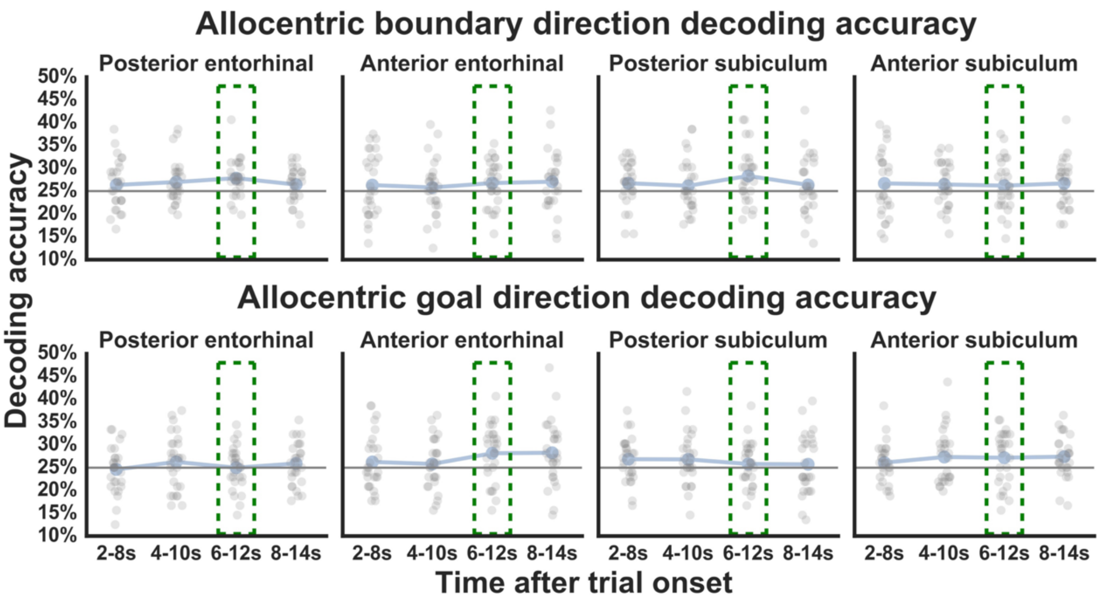

Supplementary Figure 5. Group-level decoding accuracy for allocentric boundary and allocentric goal direction as a function of time after trial onset. The horizontal grey line represents chance performance (25%) and the data points surrounded by the green ticked line represent those used in the analysis in the paper using the "Add 4-6" GLM. Individual subject's data points are represented by grey circles. Source data are provided as a Source Data file.

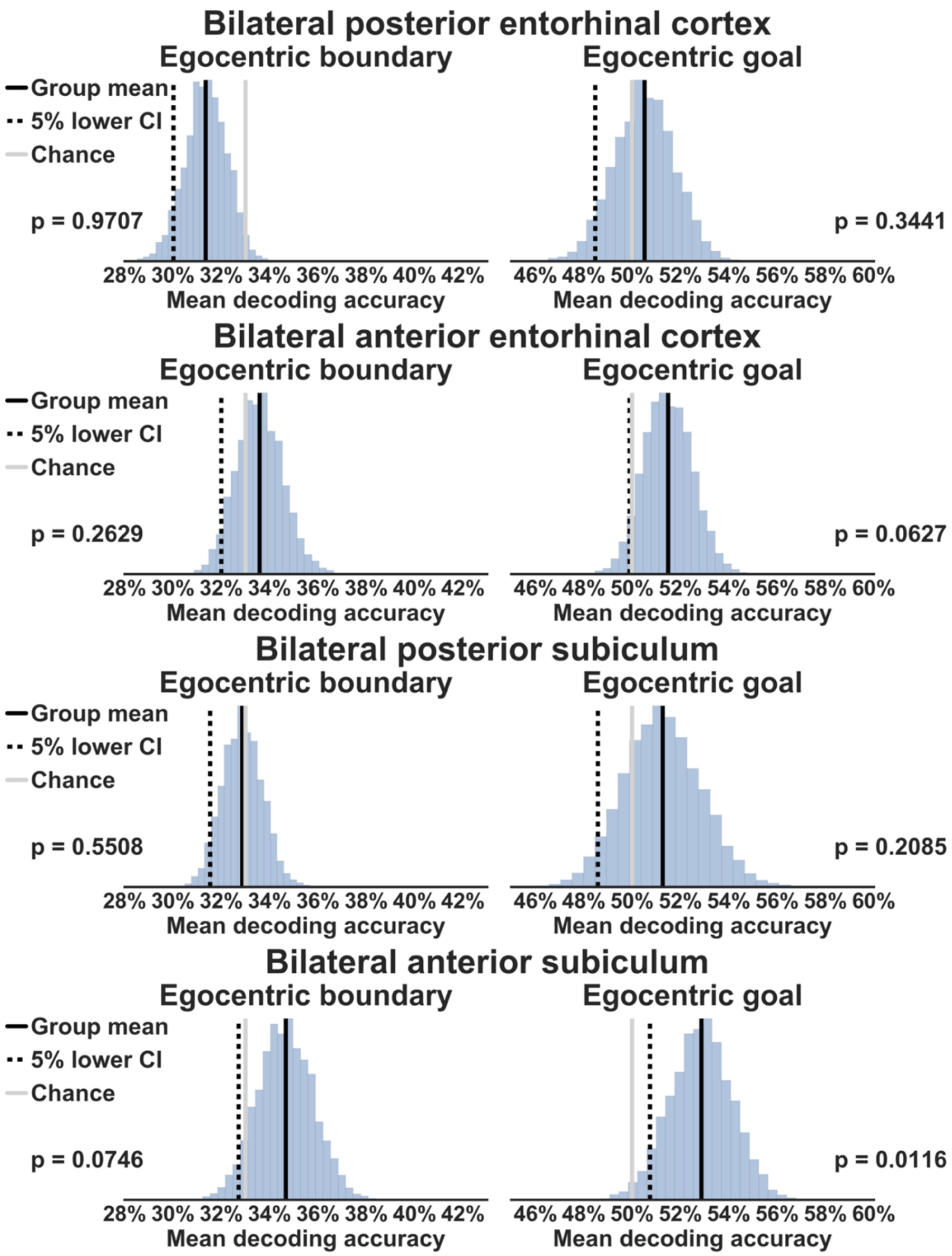

Supplementary Figure 6. Decoding of egocentric boundary and goal direction in the EC and subiculum. There was little evidence of egocentric coding in the EC and subiculum, with only the anterior subiculum showing above chance decoding for egocentric goal direction. Chance performance for egocentric boundary is 33% (boundary left, right, front of participant) and 50% for egocentric goal (left or right of path). All *p*-values were determined via non-parametric Monte Carlo significance tests. Source data are provided as a Source Data file.

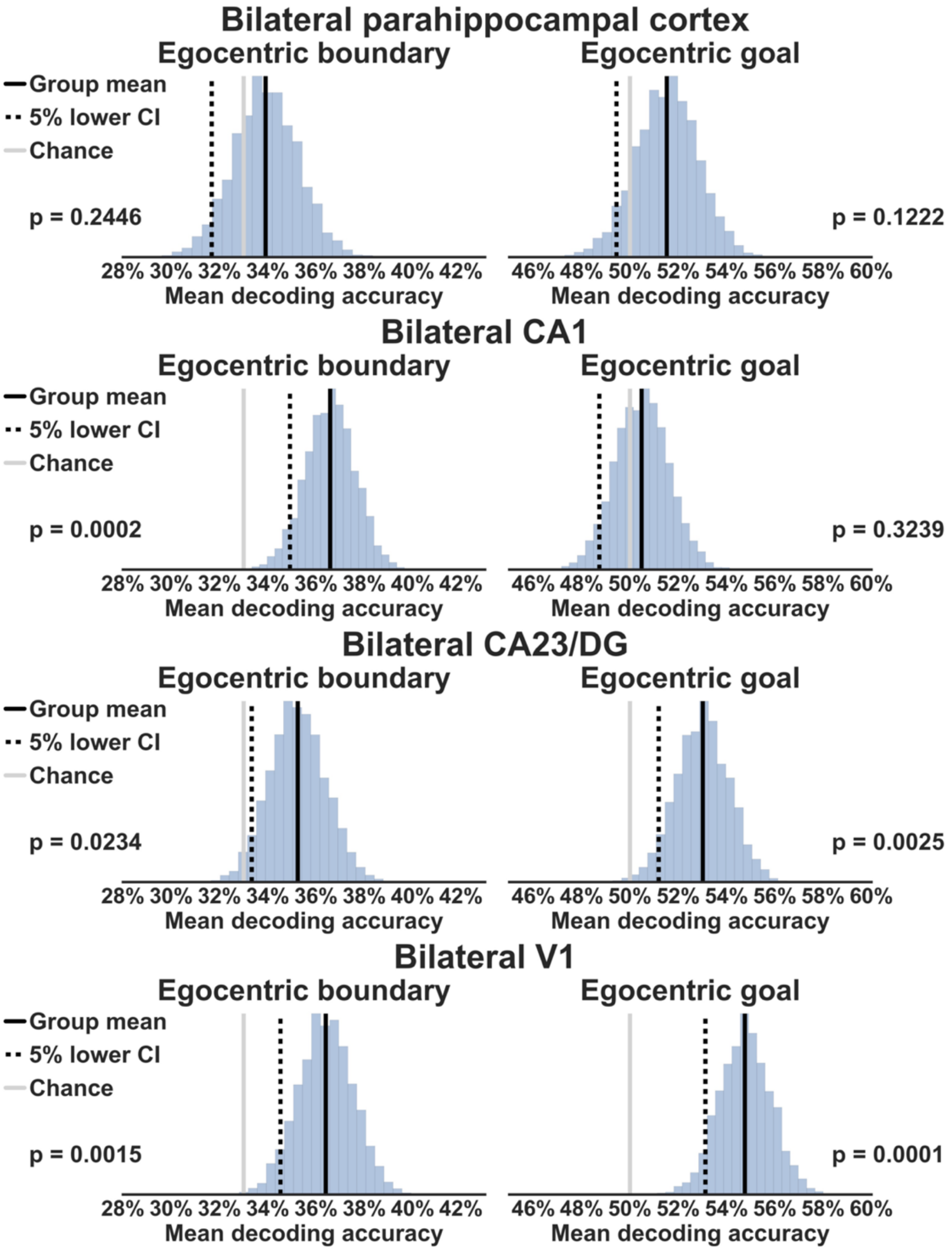

Supplementary Figure 7. Decoding of egocentric boundary and goal direction in the medial temporal lobe and V1. We were able to decode egocentric boundary direction in the CA1 and CA23/DG, whereas egocentric goal direction information was contained in CA23/DG. Chance performance for egocentric boundary is 33% (boundary left, right, front of participant) and 50% for egocentric goal (left or right of path). All *p*-values were determined via non-parametric Monte Carlo significance tests. Source data are provided as a Source Data file.

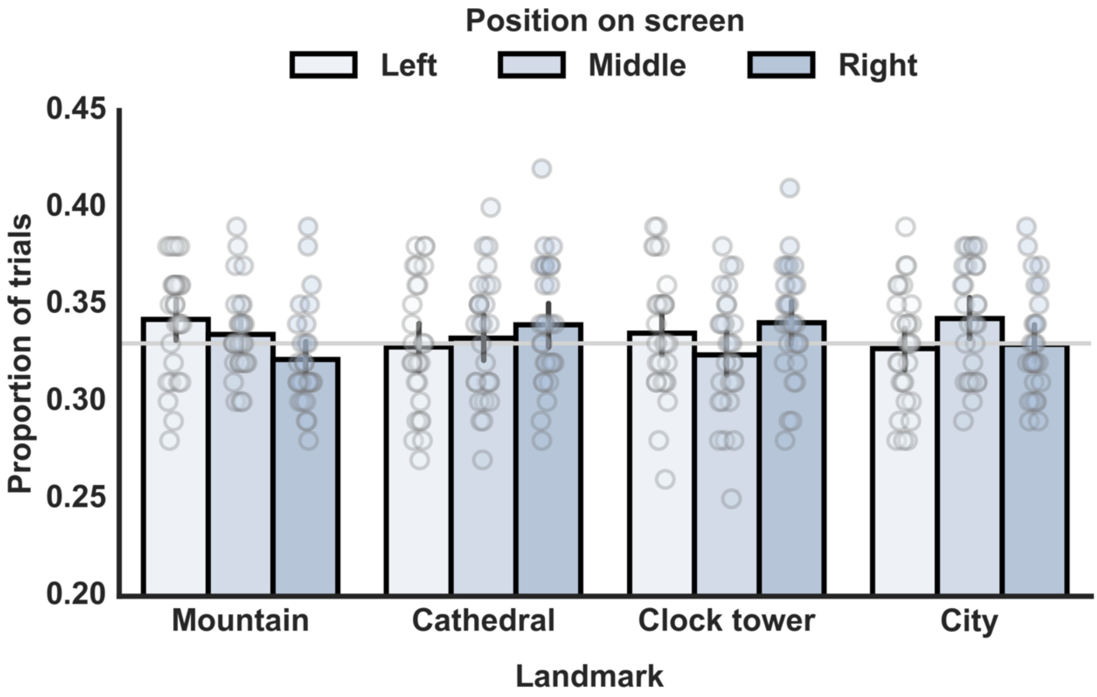

Supplementary Figure 8. Proportion of trials in which each landmark was located either to the left, middle, or right of the screen during the forced-choice response in the fMRI task (see Figure 4a in the main text). To ensure that the position of the landmark on the screen (which corresponded also to the position on the button box) did not confound our decoding results, the position of the landmarks on screen was randomised, meaning that there was no systematic relationship between landmark and screen position. Individual subject’s data points are represented by grey circles. Error bars represent 95% CI; the grey horizontal line represents chance. Source data are provided as a Source Data file.

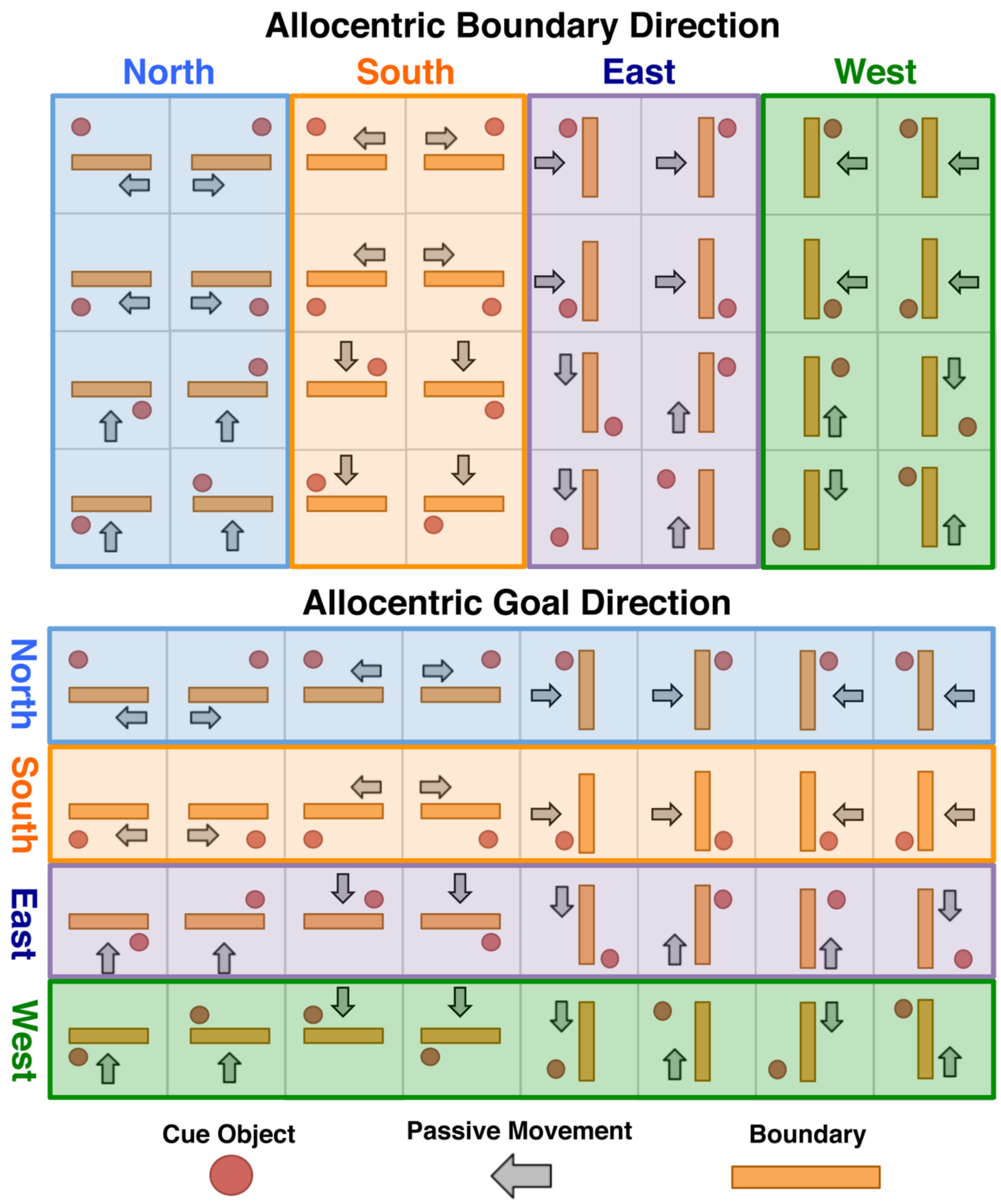

Supplementary Figure 9. Schematic of the different trials in the scanner task and the allocentric boundary and goal direction trial coding schemes. Only two (one horizontal, one vertical) of the four boundaries are displayed given that this scheme is identical for the other two boundaries. The cue object about which the participant made the goal direction judgement is represented by the filled red circle; the travel direction in the trial is indicated via the grey arrow; the boundary is reflected by the filled orange rectangles.

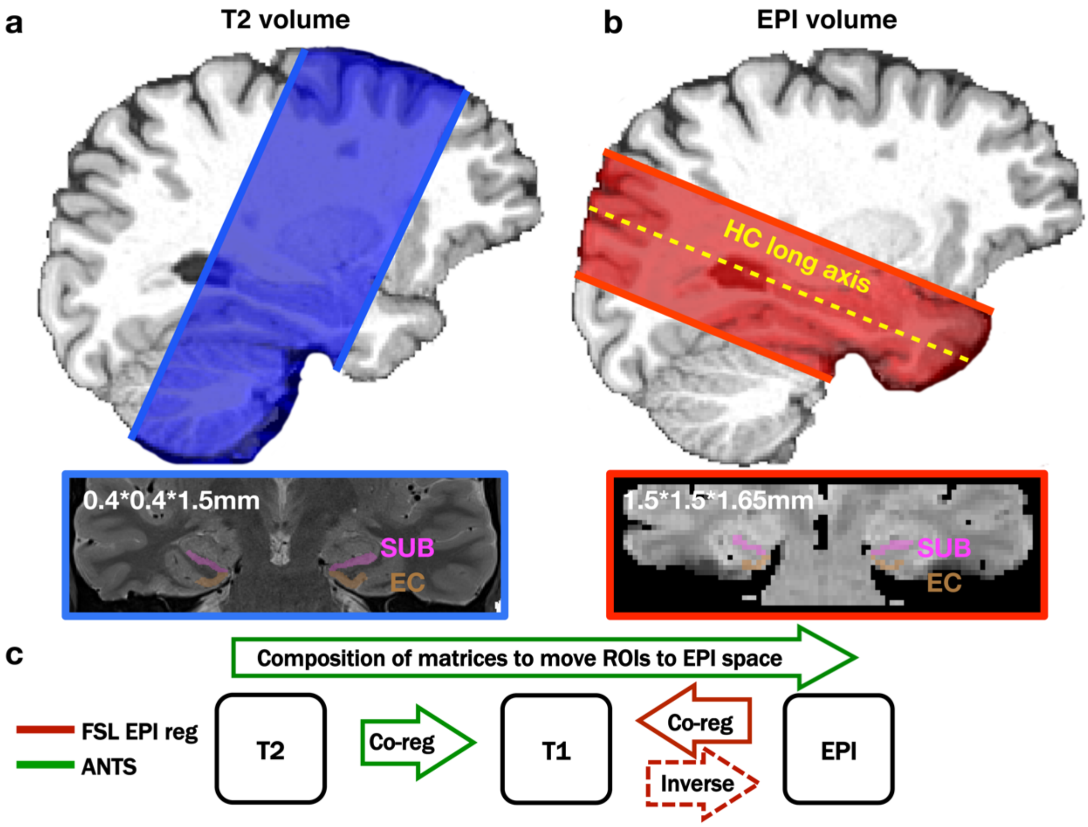

Supplementary Figure 10. Co-registration of T2 and EPI volumes. (a) Bilateral hippocampi (HC) were segmented manually on individual subject's T2-weighted images (blue). (b) Anatomical masks including the subiculum (SUB) and entorhinal cortex (EC) were then co-registered (Co-reg) with the high-resolution EPI slab aligned with the longitudinal axis of the hippocampus (red), using (c) a combination of FSL registration tool (FSL EPIreg) and ANTs.

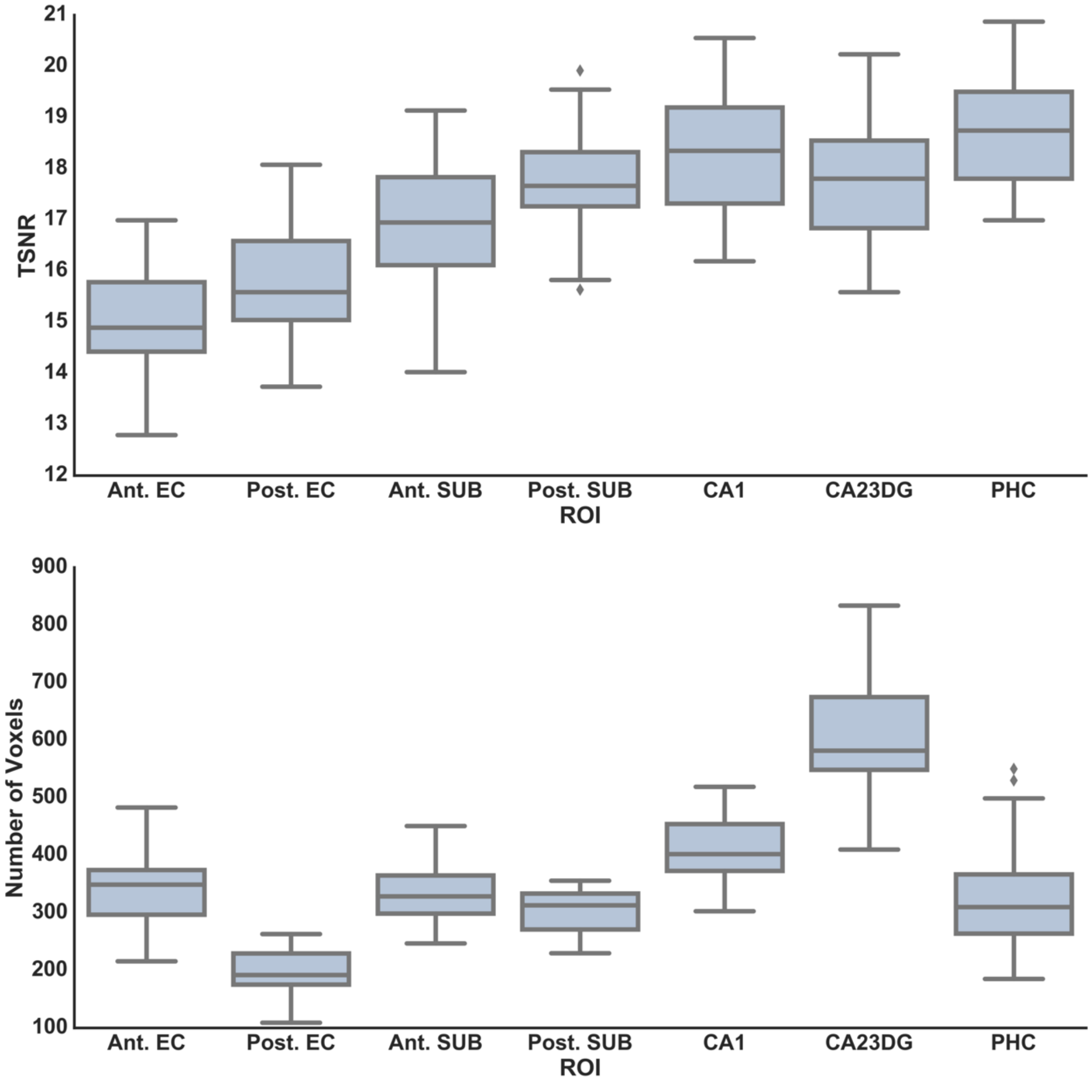

Supplementary Figure 11. Temporal signal-to-noise ratio and number of voxels for each ROI, averaged over the group (n=28). The horizontal line represents the median while the box limits represent the upper and lower quartiles; the whiskers indicate 1.5 x the interquartile range, and outliers are indicated with diamond markers. Source data are provided as a Source Data file.

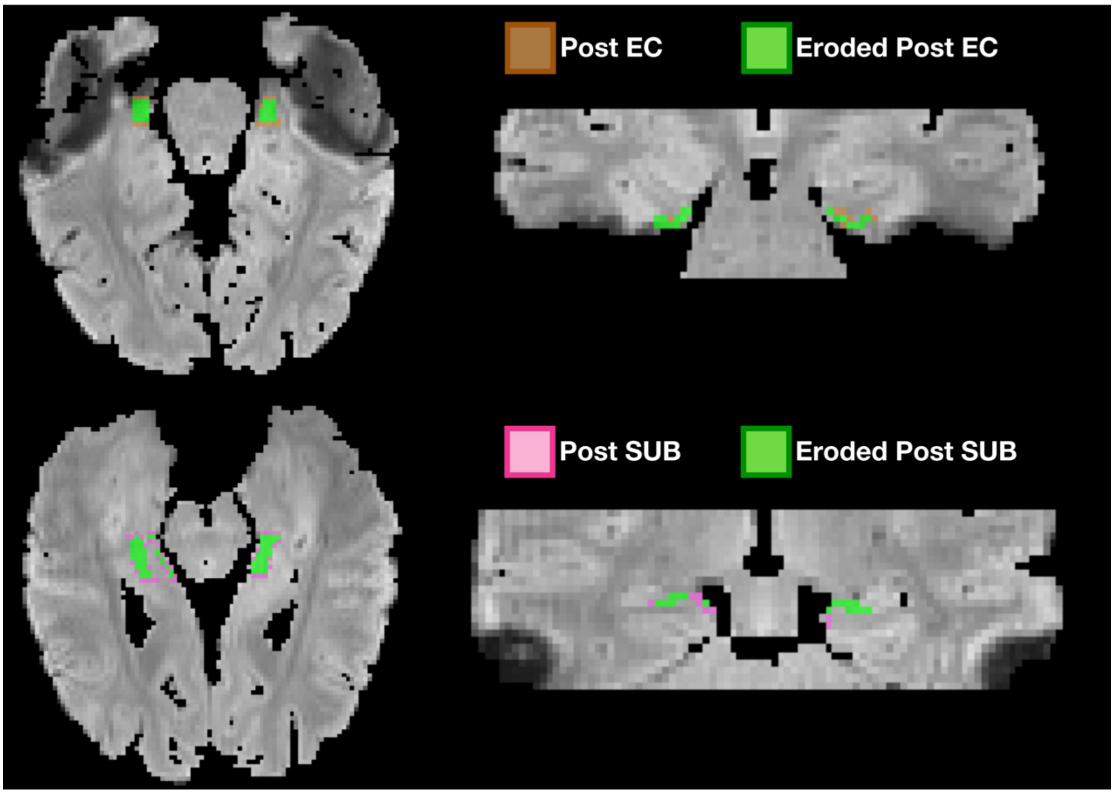

Supplementary Figure 12. Example of one participant’s eroded and original entorhinal cortex (EC) and subiculum (SUB) ROI masks rendered on the mean EPI image.

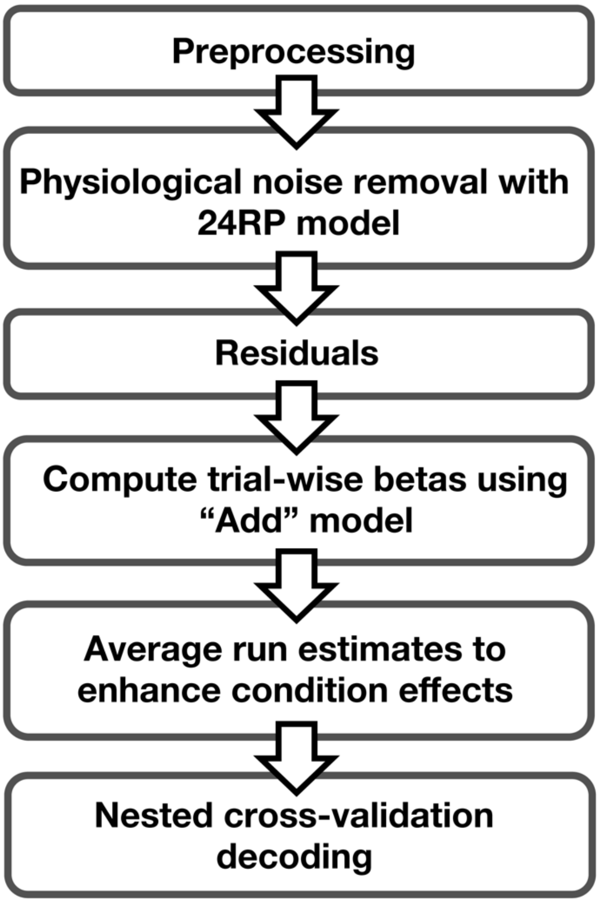

Supplementary Figure 13. Overview of fMRI analysis pipeline.

Supplementary Tables

	Same Allo. Dir.		Diff. Allo. Dir.					95% CI		
ROI	Mean	SD	Mean	SD	*t*	df	*p*	Lower	Upper	Hedges $_{gav}$
Post. EC	-0.006	0.003	-0.008	0.003	1.87	27	0.036	-0.0001	0.0034	0.52
Ant. EC	-0.008	0.002	-0.007	0.002	-0.10	27	0.462	-0.0013	0.0014	0.03
Post. Sub.	-0.007	0.003	-0.007	0.003	0.31	27	0.378	-0.0011	0.0016	0.07
Ant. Sub.	-0.007	0.003	-0.008	0.002	0.25	27	0.402	-0.0014	0.0018	0.07
CA1	-0.007	0.002	-0.007	0.002	0.87	27	0.196	-0.0006	0.0016	0.20
CA23DG	-0.007	0.002	-0.007	0.002	0.43	27	0.336	-0.0009	0.0014	0.12
PHC	-0.005	0.003	-0.005	0.003	0.36	27	0.359	-0.0012	0.0017	0.09

Supplementary Table 1. Comparison of mean representational similarity for same versus different allocentric boundary direction.

Supplementary References

1. Sarel, A., Finkelstein, A., Las, L. & Ulanovsky, N. Vectorial representation of spatial goals in the hippocampus of bats. *Science.* 355, 176–180 (2017).
2. Wang, C. *et al.* Egocentric coding of external items in the lateral entorhinal cortex. *Science.* 362, 945–949 (2018).
3. Chadwick, M. J., Jolly, A. E. J., Amos, D. P., Hassabis, D. & Spiers, H. J. A Goal Direction Signal in the Human Entorhinal / Subicular Region. *Curr. Biol.* 25, 1–6 (2015).
4. Mumford, J. A., Turner, B. O., Ashby, F. G. & Poldrack, R. A. Deconvolving BOLD activation in event-related designs for multivoxel pattern classification analyses. *Neuroimage* 59, 2636–2643 (2012).
5. Nili, H. *et al.* A Toolbox for Representational Similarity Analysis. *PLoS Comput. Biol.* 10, 10.1371/journal.pcbi.1003553 (2014).
6. Mur, M., Bandettini, P. A. & Kriegeskorte, N. Revealing representational content with pattern-information fMRI - An introductory guide. *Soc. Cogn. Affect. Neurosci.* 4, 101–109 (2009).
