# Supplementary material for: Evidence for allocentric boundary and goal direction information in the human entorhinal cortex and subiculum
Source: Nat Commun. 2019 Sep 5;10:4004. doi: 10.1038/s41467-019-11802-9 (PMC6728372; doi:10.1038/s41467-019-11802-9)
Supplement: Supplementary file 3 — Reporting Summary [file 41467_2019_11802_MOESM3_ESM.pdf]

nature research

Corresponding author(s): Jonathan Shine

Reporting Summary

Nature Research wishes to improve the reproducibility of the work that we publish. This form provides structure for consistency and transparency in reporting. For further information on Nature Research policies, see Authors & Referees and the Editorial Policy Checklist.

Statistical parameters

When statistical analyses are reported, confirm that the following items are present in the relevant location (e.g. figure legend, table legend, main text, or Methods section).

n/a	Confirmed	
☐	☒	The exact sample size (n) for each experimental group/condition, given as a discrete number and unit of measurement
☐	☒	An indication of whether measurements were taken from distinct samples or whether the same sample was measured repeatedly
☐	☒	The statistical test(s) used AND whether they are one- or two-sided<br>*Only common tests should be described solely by name; describe more complex techniques in the Methods section.*
☒	☐	A description of all covariates tested
☐	☒	A description of any assumptions or corrections, such as tests of normality and adjustment for multiple comparisons
☐	☒	A full description of the statistics including central tendency (e.g. means) or other basic estimates (e.g. regression coefficient) AND variation (e.g. standard deviation) or associated estimates of uncertainty (e.g. confidence intervals)
☐	☒	For null hypothesis testing, the test statistic (e.g. F, t, r) with confidence intervals, effect sizes, degrees of freedom and P value noted<br>*Give P values as exact values whenever suitable.*
☒	☐	For Bayesian analysis, information on the choice of priors and Markov chain Monte Carlo settings
☒	☐	For hierarchical and complex designs, identification of the appropriate level for tests and full reporting of outcomes
☐	☒	Estimates of effect sizes (e.g. Cohen's d, Pearson's r), indicating how they were calculated
☐	☒	Clearly defined error bars<br>*State explicitly what error bars represent (e.g. SD, SE, CI)*

Our web collection on statistics for biologists may be useful.

Software and code

Policy information about availability of computer code

Data collection	WorldViz Vizard 5.1 Virtual Reality Software (WorldViz LLC, http://www.worldviz.com) was used to create the virtual environment, train the participants, and run the fMRI scan task.
Data analysis	For the analysis of the imaging data we used: SPM12, FSL5.0.6, Advanced Normalisation Tools (2.1), and Nipype 0.11. To analyse and plot the group decoding accuracies we used numpy 1.10.3, pandas 0.21, python 3.5.2, Sci-kitlearn 0.19.1, matplotlib 2.0.1, seaborn 0.7.1, and SPSS (version 21.0).

For manuscripts utilizing custom algorithms or software that are central to the research but not yet described in published literature, software must be made available to editors/reviewers upon request. We strongly encourage code deposition in a community repository (e.g. GitHub). See the Nature Research guidelines for submitting code & software for further information.

Data

Policy information about availability of data

All manuscripts must include a data availability statement. This statement should provide the following information, where applicable:

- Accession codes, unique identifiers, or web links for publicly available datasets
- A list of figures that have associated raw data
- A description of any restrictions on data availability

The data that support the findings of this study are available from the corresponding author upon reasonable request.

Field-specific reporting

Please select the best fit for your research. If you are not sure, read the appropriate sections before making your selection.

☒ Life sciences ☐ Behavioural & social sciences ☐ Ecological, evolutionary & environmental sciences

For a reference copy of the document with all sections, see nature.com/authors/policies/ReportingSummary-flat.pdf

Life sciences study design

All studies must disclose on these points even when the disclosure is negative.

Sample size

Our sample size (n = 28 after three participants were excluded) was chosen to be consistent with previous spatial navigation tasks in which participants have been required to imagine goal directions (n = 24; Bellmund et al., 2016), navigate to goal locations (n = 23; Sherrill et al., 2013), and imagine different facing directions in a virtual environment (n = 16; Chadwick et al., 2015).

References:
Chadwick, M. J., Jolly, A. E. J., Amos, D. P., Hassabis, D. & Spiers, H. J. A Goal Direction Signal in the Human Entorhinal / Subicular Region. Curr. Biol. 25, 1–6 (2015).
Sherrill, K. R. et al. Hippocampus and Retrosplenial Cortex Combine Path Integration Signals for Successful Navigation. J. Neurosci. 33, 19304–19313 (2013).
Bellmund, J. L. S., Deuker, L., Schroeder, T. N. & Doeller, C. F. Grid-cell representations in mental simulation. Elife 5, (2016).

Data exclusions

Three participants were not included in the analysis. This was due to movement artifacts in the T2 image that prevented us from segmenting our regions of interest.

Replication

We did not attempt to replicate the findings of the study.

Randomization

The study did not use different experimental groups and therefore we did not need to randomly allocate participants.

Blinding

We used a repeated measures design in which all participants completed all conditions of the experiment, and there were no separate groups. Blinding, therefore, was not relevant for this study.

Reporting for specific materials, systems and methods

Materials & experimental systems

n/a	Involved in the study	
☐	☐	Unique biological materials
☐	☐	Antibodies
☐	☐	Eukaryotic cell lines
☐	☐	Palaeontology
☐	☐	Animals and other organisms
☐	☒	Human research participants

Methods

n/a	Involved in the study	
☐	☐	ChIP-seq
☐	☐	Flow cytometry
☐	☒	MRI-based neuroimaging

Unique biological materials

Policy information about availability of materials

Obtaining unique materials	*Describe any restrictions on the availability of unique materials OR confirm that all unique materials used are readily available from the authors or from standard commercial sources (and specify these sources).*

Antibodies

Antibodies used	*Describe all antibodies used in the study; as applicable, provide supplier name, catalog number, clone name, and lot number.*
Validation	*Describe the validation of each primary antibody for the species and application, noting any validation statements on the manufacturer's website, relevant citations, antibody profiles in online databases, or data provided in the manuscript.*

Eukaryotic cell lines

Policy information about cell lines

Cell line source(s)	*State the source of each cell line used.*
Authentication	*Describe the authentication procedures for each cell line used OR declare that none of the cell lines used were authenticated.*
Mycoplasma contamination	*Confirm that all cell lines tested negative for mycoplasma contamination OR describe the results of the testing for mycoplasma contamination OR declare that the cell lines were not tested for mycoplasma contamination.*
Commonly misidentified lines (See ICLAC register)	*Name any commonly misidentified cell lines used in the study and provide a rationale for their use.*

Palaeontology

Specimen provenance	*Provide provenance information for specimens and describe permits that were obtained for the work (including the name of the issuing authority, the date of issue, and any identifying information).*
Specimen deposition	*Indicate where the specimens have been deposited to permit free access by other researchers.*
Dating methods	*If new dates are provided, describe how they were obtained (e.g. collection, storage, sample pretreatment and measurement), where they were obtained (i.e. lab name), the calibration program and the protocol for quality assurance OR state that no new dates are provided.*

☐ Tick this box to confirm that the raw and calibrated dates are available in the paper or in Supplementary Information.

Animals and other organisms

Policy information about studies involving animals; ARRIVE guidelines recommended for reporting animal research

Laboratory animals	*For laboratory animals, report species, strain, sex and age OR state that the study did not involve laboratory animals.*
Wild animals	*Provide details on animals observed in or captured in the field; report species, sex and age where possible. Describe how animals were caught and transported and what happened to captive animals after the study (if killed, explain why and describe method; if released, say where and when) OR state that the study did not involve wild animals.*
Field-collected samples	*For laboratory work with field-collected samples, describe all relevant parameters such as housing, maintenance, temperature, photoperiod and end-of-experiment protocol OR state that the study did not involve samples collected from the field.*

Human research participants

Policy information about studies involving human research participants

Population characteristics	31 right-handed, young healthy adults (13 female; mean age 26.12 years, range = 20 – 33 years) participated in the experiment.
Recruitment	Participants were recruited via phone from a database of individuals with no MR contraindications.

ChIP-seq

Data deposition

☐ Confirm that both raw and final processed data have been deposited in a public database such as GEO.

☐ Confirm that you have deposited or provided access to graph files (e.g. BED files) for the called peaks.

Data access links<br>*May remain private before publication.*	*For "Initial submission" or "Revised version" documents, provide reviewer access links. For your "Final submission" document, provide a link to the deposited data.*
Files in database submission	*Provide a list of all files available in the database submission.*
Genome browser session<br>(e.g. UCSC)	*Provide a link to an anonymized genome browser session for "Initial submission" and "Revised version" documents only, to enable peer review. Write "no longer applicable" for "Final submission" documents.*

Methodology

Replicates	*Describe the experimental replicates, specifying number, type and replicate agreement.*
Sequencing depth	*Describe the sequencing depth for each experiment, providing the total number of reads, uniquely mapped reads, length of reads and whether they were paired- or single-end.*
Antibodies	*Describe the antibodies used for the ChIP-seq experiments; as applicable, provide supplier name, catalog number, clone name, and lot number.*
Peak calling parameters	*Specify the command line program and parameters used for read mapping and peak calling, including the ChIP, control and index files used.*
Data quality	*Describe the methods used to ensure data quality in full detail, including how many peaks are at FDR 5% and above 5-fold enrichment.*
Software	*Describe the software used to collect and analyze the ChIP-seq data. For custom code that has been deposited into a community repository, provide accession details.*

Flow Cytometry

Plots

Confirm that:

☐ The axis labels state the marker and fluorochrome used (e.g. CD4-FITC).

☐ The axis scales are clearly visible. Include numbers along axes only for bottom left plot of group (a 'group' is an analysis of identical markers).

☐ All plots are contour plots with outliers or pseudocolor plots.

☐ A numerical value for number of cells or percentage (with statistics) is provided.

Methodology

Sample preparation	*Describe the sample preparation, detailing the biological source of the cells and any tissue processing steps used.*
Instrument	*Identify the instrument used for data collection, specifying make and model number.*
Software	*Describe the software used to collect and analyze the flow cytometry data. For custom code that has been deposited into a community repository, provide accession details.*
Cell population abundance	*Describe the abundance of the relevant cell populations within post-sort fractions, providing details on the purity of the samples and how it was determined.*
Gating strategy	*Describe the gating strategy used for all relevant experiments, specifying the preliminary FSC/SSC gates of the starting cell population, indicating where boundaries between "positive" and "negative" staining cell populations are defined.*

☐ Tick this box to confirm that a figure exemplifying the gating strategy is provided in the Supplementary Information.

Magnetic resonance imaging

Experimental design

Design type	The study used an event-related design.

Design specifications

The experiment comprised three separate runs of 96 trials. Each trial lasted eight seconds and there was a one second mean inter-trial interval.

Behavioral performance measures

Mean accuracy (i.e., proportion of correct responses) and reaction time were used to assess participant performance in both the learning and fMRI task.

Acquisition

Imaging type(s)

The study used functional imaging.

Field strength

3.0 Tesla.

Sequence & imaging parameters

Imaging data were acquired using a 3T SIEMENS (Erlangen, Germany) Magnetom Prisma scanner, with a 64-channel phased array head coil. Scans comprised a whole-head, three-dimensional structural T1-weighted anatomical image with 1 mm isotropic resolution (TR/TE/Inversion time = 2500/2.82/1100 ms; flip-angle = 7 degrees; FOV = 256 × 256 mm2; 192 slices; GRAPPA acceleration factor 2); a high resolution moderately T2-weighted structural image comprising the hippocampus and EC acquired perpendicular to the long axis of the hippocampus using a turbo-spin-echo sequence (in-plane resolution = 0.4 × 0.4 mm2, slice-thickness = 1.5 mm; TR/TE = 4540ms/44ms; FOV = 224 × 224 mm2; 32 slices); gradient echo field maps (in-plane resolution = 1.6 × 1.6 mm2; slice-thickness = 2 mm; TR/TE1/TE2 = 720/4.92/7.38 ms; flip-angle = 60 degrees; FOV = 220 × 220 mm2; 72 slices), and three runs (445 volumes each) of T2*-weighted functional images acquired with a partial-volume echo-planar imaging sequence, aligned with the long axis of the hippocampus (in-plane resolution = 1.5 × 1.5 mm2, slice-thickness = 1.5 mm + 10% gap; TR/TE = 2000/30 ms; flip angle = 90 degrees; FOV = 192 × 192 mm2; 26 slices; GRAPPA acceleration factor 2).

Area of acquisition

Due to the high-resolution of the functional data, data acquisition was restricted to an EPI slab encompassing the hippocampus and entorhinal cortex. The functional volume was aligned with the mid-point of the long axis of the hippocampus.

Diffusion MRI ☐ Used ☒ Not used

Preprocessing

Preprocessing software

- SPM 12: Bias correction and segmentation, Realign
- FSL 5.0.6: Registration of EPI to T1 weighted anatomical image with field map correction (epireg)
- ANTS 2.1: Registration of T1 weighted anatomical image to T2 weighted image
- Nipype 0.11: Preprocessing pipeline

Normalization

N/A

Normalization template

N/A

Noise and artifact removal

Prior to the decoding analysis, movement parameters obtained from the realignment of the functional images were regressed out of the data. Here, we included 24 regressors in the model, reflecting the realignment parameters, their derivatives, their squares, and their square derivatives.

Volume censoring

Volume censoring was not implemented in the study.

Statistical modeling & inference

Model type and settings

Each of the 96 trials per run was modelled separately in the analysis. To reduce the possible influence of visual information in our decoding analysis, we analysed the portion of data corresponding to the period of the trial after the passive movement ended during which there was no visual input (i.e., a black screen) and was therefore matched across different allocentric boundary/goal directions. To account for the lag of the haemodynamic response function, we analysed the volumes occurring 4-6 seconds after the onset of this period of the stationary period of the trial, and averaged the data over the next consecutive three volumes (Mourão-Miranda et al., 2006).
To enhance the signal corresponding to the allocentric condition of interest whilst maintaining the voxel space, we created an average over the three runs by first ordering the trials in each run according to the condition to-be-decoded (allocentric boundary or allocentric goal). The rationale here was to strengthen the condition of interest, whilst weakening any signal associated with other conditions (e.g., head direction). This trial-averaging resulted in 96 samples per participant, balanced equally across North, South, East and West directions for allocentric boundary and allocentric goal conditions.

References:
Mourão-Miranda, J., Reynaud, E., McGlone, F., Calvert, G. & Brammer, M. The impact of temporal compression and space selection on SVM analysis of single-subject and multi-subject fMRI data. Neuroimage 33, 1055–1065 (2006).

Effect(s) tested

We tested to see whether mean decoding accuracy was significantly greater than chance for allocentric boundary and allocentric goal direction in the entorhinal cortex and subiculum.

Given our apriori predictions regarding differences according to anterior versus posterior regions of the entorhinal cortex and subiculum, we submitted our group decoding accuracies to a repeated-measures ANOVA comprising the factors i) ROI (entorhinal cortex, subiculum) ii) Anterior/posterior section, and iii) Condition (allocentric boundary direction, allocentric goal direction).

Specify type of analysis: ☐ Whole brain ☒ ROI-based ☐ Both

Anatomical location(s)

Anatomical ROIs comprising the entorhinal cortex, subiculum, CA1, CA23DG and parahippocampus were identified via manual segmentation of individual participants' T2 images following established protocol (Berron et al., 2017).

References:

Berron, D. et al. A protocol for manual segmentation of medial temporal lobe subregions in 7 Tesla MRI. NeuroImage Clin. 15, 466–482 (2017).

Statistic type for inference
(See Eklund et al. 2016)

N/A

Correction

Where appropiate, results were Bonferroni-corrected for multiple comparisons.

Models & analysis

n/a	Involved in the study
☒	☐ Functional and/or effective connectivity
☒	☐ Graph analysis
☐	☒ Multivariate modeling or predictive analysis

Multivariate modeling and predictive analysis

A support vector classifier with L2 regularization was used for the decoding of different allocentric directions. The regularization strength was determined by adjusting the C hyperparameter. In order to follow decoding best practices, we used nested cross-validation to estimate the best C hyperparameter and obtained a cross-validated estimate of the classifier accuracy with three outer folds using 20% of the data as test set in each fold. The best hyperparameter was chosen within the inner nested cross-validated fold, using a grid search with possible values in the range of 1 to 10ˆ3 in steps of power of 10. All decoding analyses were conducted in the participants' native EPI space.

For the separate ROIs, we obtained the mean decoding score per participant over the three-folds of the cross-validation. We then used the bias-corrected and accelerated boot-strap (BCa) (Efron & Tibshirani, 1994) to sample from these values 10,000 times to obtain the distribution of our group-level decoding accuracy. Non-parametric Monte Carlo significance tests were used to generate a p-value based on the distribution of our data, where we first subtracted the group mean decoding accuracy from each participant’s decoding score, before adding chance performance (i.e., 25%). This had the effect of shifting the distribution of our group’s decoding scores to around chance performance, and we then again used the BCa (with 10,000 samples) with these values to generate our null distribution. The one-tailed p-value was calculated by counting the number of times the boot-strap null mean exceeded our observed group-level decoding score, adding one to this value, and dividing this value by the number of samples (i.e., 10,000) plus one.

References:

Efron, B. & Tibshirani, R. J. An Introduction to the Bootstrap - CRC Press Book. Chapman and Hall/CRC 436 (1994). Available at: https://www.crcpress.com/An-Introduction-to-the-Bootstrap/Efron-Tibshirani/p/book/9780412042317
